# Transactions of the Medical Society of New York. Vol. 1835

**Published:** 1835-07-01

**Authors:** 


### TRANSACTIONS OF THE MEDICAL SOCIETY OF NEW YORK. VOL. I. 1832-3.

WE have not been able to procure the above work, and must therefore take the following short analysis of it from one of our transatlantic contemporaries, (the Baltimore Medical Journal,) now, we grieve to say, no more. We find, by a notice at the end of the second volume of the above work, that the labours of the industrious and learned editor, Dr. Gedings, have terminated, as far as journalism is concerned. We hope the real cause of this premature decease of our contemporary is the one assigned—want of leisure on the part of the Editor—not want of patronage on the part of the public. Periodical literature, however, like every other species of intellectual commerce, is fast overflowing, and individual losses as well as mortification must be the inevitable consequence. Journals, and more especially REVIEWS, are peculiarly liable to this commercial glut and stagnation. It is a difficult thing for one work to supplant another, unless great negligence or inability is displayed on one side; and it is still more difficult to create a demand in the public market for the same thing twice over—which the review of a book must be, in two journals. Reviews *must* now be analytical, rather than critical. Reviewers, like ministers, *must* run in a groove—and if they get out of that groove, they are lost. Time past has furnished illustrations, and time to come will exhibit more. But this is a digression.

On the 7th of February, 1832, the Medical Society of the state of New York, while in session in Albany, resolved to publish its transactions in a permanent form. The publication is destined to embrace successful prize dissertations, addresses made from year to year by the President, the medical topographical reports, made by the respective County Medical Societies, proceedings of the Society, &c. &c.

The committee charged with the execution of this plan have published the volume before us. It is very creditable to the professional zeal of the New York Medical Society.

The initial article of part one, is a prize dissertation on delirium tremens, by James Conquest Cross, M. D. of Lexington, Kentucky.

After treating on the literary history, name, etiology, and general character of the disease, Dr. Cross considers its varieties. He enumerates no less than four, viz.—1, The Sthenic; which consists in vasculur irritation, or a condition, which doubtless predisposes to, and frequently terminates in, inflammation. 2. The Hypersthenic; this consists in inflammation. 3. The Asthenic; consisting in nervous irritation. 4. The Bilious; which consists in the supervention of delirium tremens upon bilious fever. Dr. C. does not relish the praise which journalists have bestowed on the essay of Dr. Coates, on delirium tremens. He thinks Dr. Coates has not fairly represented the disease. The remarks of this gentleman, that *pain* has never been observed by him in delirium tremens, either in the head, or in any other part of the body; that *increased heat*, like pain, has never occurred in the cases under his observation, unless where it already existed, as the product of a previously existing malady; and that the pulse, according to his observations, is generally weak, are considered by Dr. Cross so unfaithful an account of delirium tremens, as to "betray a manifest and reprehensible unwillingness to admit, as attributes of this disease, any of those symptoms that denote the existence of sthenic or hypersthenic action." Dr. Cross has here levelled six pages of criticism at Dr. Coates' essay. He admits it possesses intrinsic excellence, but, "it unfortunately happens, that his views, and those of that very respectable physician, (Dr. Coates,) wage against each other a war of mutual extermination!" Each of these authors will find in the other a formidable combatant, and we hope their discussion will more fully elucidate the disease. Dr. Cross next treats of the prognosis, diagnosis, and necroscopic reports. Twenty-six pages are then devoted to a discussion on the proximate cause of delirium tremens. This, he attempts to demonstrate, consists originally either in vascular irritation, actual inflammation, or nervous irritation of the liver, and, subsequently, of the brain.

*Treatment of Delirium Tremens.*—On the use of the lancet, Dr. Cross says—the necessity for either general or local blood-letting does not frequently occur in *sthenic delirium tremens.* In *hypersthenic delirium tremens,* the indication is much plainer, and the propriety of the general detraction of blood is frequently imperious and necessary. The following remarks of Dr. Coates, when applied to *asthenic* delirium tremens, meet with the author's fullest approbation. "My own experience leads me distinctly to say, that the lancet does not appear to have the least discoverable effect, positive or negative, upon the delirium, and that it actually does harm to the patient, by diminishing his strength, an item highly necessary to the cure." Dr. Cross has never ventured on the use of the lancet in *bilious delirium tremens;* he has occasionally detracted blood locally, not however without its propriety being clearly indicated.

*Of Emetics.*—In sthenic delirium tremens, it will be rarely necessary to use emetics. They should be wholly proscribed in the hypersthenic. It is in the asthenic and bilious varieties that our emetics are strongly indicated and particularly useful.

*Cathartics.*—No variety of delirium tremens has ever deterred the author from using cathartics; although they are beneficial, they are far from being so to an equal extent, in every species. He prefers calomel, aloes, and rhubarb.

*Opium.*—Twenty-five pages are given to a consideration of this celebrated remedy. In *sthenic* delirium tremens, after the bowels have been freely opened and the liver stimulated to the production of a copious discharge of bile, by the action of a dose of calomel, aloes and rhubarb, if the pulse should be soft and compressible, the author commences the exhibition of calomel and opium conjoined. Twenty grains of the former and three of the latter is his ordinary dose at the commencement. In three hours the opium alone is repeated, and in three hours more the same quantity of calomel and opium. In two or three hours after the exhibition of the last dose, fifteen grains of aloes, with the same quantity of rhubarb are given. This simple plan has never failed but in one single instance. In *hypersthenic* d. tremens, before using opium, the lancet and cathartics should be employed until the pulse becomes soft and compressible. Two or three days will be required for this preliminary treatment in a majority of cases. It will moderate the primitive inflammation of the stomach, and the secondary inflammation of the brain, so as to enable the opium and calomel to act efficiently.

In this variety, the author rarely commences with less than four grains of opium, and half a drachm of calomel.

In *asthenic* d. tremens the author purges previous to the administration of opium. If a suitable dose of calomel, aloes and rhubarb, does not operate, he recommends one drop of croton oil every two hours. In this variety, as the irritation is exclusively nervous in its character, the necessity of adding calomel is not so imperious. Three grains of opium every three hours, are recommended, by the author, after the preliminary treatment. In the early stage of *bilious* d. tremens, the author purges as before with calomel, aloes and rhubarb. On the pulse becoming soft and compressible, he uses the following prescription, to excite the liver, to extinguish nervous irritation, and to support the strength of the system, viz.

*Calomel*, gr. v.

*Syrup of Morphine*, ℥ij.

*Sub. Carb. Ammo.* gr. iv.

This dose is given, according to circumstances, every three, four, or five hours.

Dr. Cross next makes remarks on camphor, henbane, atropa belladonna, tela araneæ, hops, blisters, cold affusions, pediluvia, ardent spirits and moral treatment. In the hypersthenic variety, he has seen a blister on the epigastrium, greatly diminish cerebral determination and excitement. The precordial oppression in the asthenic and bilious varieties, receives signal relief from blisters, used as auxiliaries to emetics and cathartics. Respecting ardent spirits, Dr. C. says: "It should enter as a precept into the science of medical ethics, that ardent spirits should never be administered as a remediate agent in the treatment of any disease, unless it is imperiously required by emergencies, and in circumstances too, where no substitute can be employed of great efficiency." He deprecates the use of this remedy in any variety of delirium tremens, in strong terms.

In the adynamic stage of this disease, Dr. C. has used with most advantage, the subcarbonate of ammonia, sulphate of quinine, sulphuric ether, assafœtida and opium. The remainder of Dr. C's. dissertation contains cases.

Article II. This is an address on Puerperal Fever, delivered before the Medical Society, by Jonathan Eights, M.D. of Albany, its president, (1832.) Dr. E. regards puerperal fever as a pure inflammatory affection in its original state. He thinks it doubtful whether the disease ever appears as an epidemic; where it seems to assume this character, it is under the influence of some other prevailing disease.

Admitting the disease to be inflammatory, Dr. E. seeks for "its location or primary seat." He determines it is in the uterus, and that the contiguous parts become affected from this source. His argument is drawn from post-mortem examinations made by order of government, in the lying-in institution in Vienna. The result of this inquiry was, that almost in every case examined after death, there were evident marks that the primary disease was in the uterus.

"From the result of the above investigations," says Dr. E. "we may come to the conclusion, that when the contents of the gravid uterus have been expelled in parturition, the orifices of the uterine veins, where the placenta had been attached, are left open, and most probably a communication is indirectly formed between the venous system and the atmospheric air: such a condition of the uterine veins, in consequence of the separation of the placenta, must be favorable to the production of inflammation, which, once excited, is seldom limited to the orifices of the vessels, but extends, with more or less rapidity, along the continuous membranes of the veins of the uterus, until a general affection is produced. The muscular substance of the uterus also becomes affected from this local source; this is communicated to the peritoneal covering, and the usual symptoms of puerperal fever will ensue."

The following remarks of Dr. E. are important and judicious.

"There is a disease, however, not unfrequently met with in the parturient state, which, from its symptoms, so closely resembles puerperal fever, as often to deceive a critical observer. It comes on a few days after delivery, with diffused pain and tenderness over the whole abdomen, and with a pulse somewhat accelerated. I have never observed it unless after the exhibition of a cathartic, acting too freely, at other times not sufficiently, producing severe gripings, and an irritable state of the bowels. The usual remedies for inflammation, viz. bleeding, purging, &c. do much injury, and if persisted in will prove fatal to the patient. Instances are on record of several who have died from active depletion, and upon examination, not a vestige of inflammation has been discovered, either in the uterus or peritoneum, and only a few ounces of colorless fluid have been found in the cavity of the abdomen. It is most frequent among women who are of delicate health and sensitive nerves.

This attack, besides the operation of a cathartic, may originate from severe and protracted after-pains passing into a permanent state, and, not unfrequently, no evident cause can be assigned.

The pulse, although somewhat quickened, is soft and feeble, and often perfectly natural; the skin remains cool, and the tongue clean, and no tumefaction or enlargement of the abdomen is discovered. These cases are not of themselves dangerous, provided the nature of them be not mistaken; nor improper remedies employed. The compound powder of ipecacuanha, warm fomentations, emollient injections, frictions over the abdomen, with anodyne liniment, in every instance relieve it."

The causes alluded to here, are analogous to those referred to by Marshall Hall, in the section on irritation, in his "researches, &c. on the loss of blood." Dr. E.'s remarks on the treatment of puerperal fever, refer to blood-letting, cathartics, fomentations, blisters, diaphoretics, calomel, oil of turpentine and emetics; the two last he condemns.

ARTICLE VI. Is an annual address on Asiatic Cholera, by Thomas Spencer, M.D. President of the Society, (1832.) The following recapitulation exhibits Dr. Spencer's view on the pathology of the several stages of cholera:—

*First State.*—1. The epidemic influence being thrown upon the exhalent tissues of the small intestines, renders it highly susceptible to the action of irritants, so that the imperfectly digested food of mild laxatives often excite profuse evacuations, and there is thus produced a disposition to violent disease from the common exciting causes of diarrhœa.

2. The stomach being weakened in its function, digestion is imperfectly performed, and at times almost suspended.

3. The liver responding to the stomach from its habitual sympathetic relations with that organ in health, falls into a state of torpor, and bile is no longer secreted.

*Second Stage.*—1. The disease essentially consists, in this stage, in a determination of fluids to the inner surface of the small intestines, diverting the respiratory, perspiratory, and urinous discharges, with their neutral salts, from their usual channels ; *and discharging them through the intestinal exhalents,* rapidly emptying the bloodvessels of their contents, and changing the relative proportions of the remnant of circulating fluids.

2. That the failure of the functions of the heart, lungs, capillary circulation, and various secretions, results from direct depletion, depriving those organs of their accustomed stimulus.

3. The absorbent system is rapidly taking up the adipose and waste parts of the body, to supply the failing resources of the heart, and thus results the rapid emaciation.

4. The spasms of the voluntary muscles, and those drawn into contractions in the act of vomiting, by compressing the intestinal exhalents, tend to arrest the discharges ; and by aiding the return of the venous circulation, stimulate the heart to redoubled exertion, giving a centrifugal direction to the circulation, thereby making a metastasis of the exhalation from the inner surface of the bowels to the skin.

5. That a striking analogy exists between this disease and hemorrhage, differing only in its effects upon the constitution, from the circumstance of its changing the relative proportions of the ingredients of the blood.

*Third Stage.*—1. Collapse consists in direct debility and failure of the functions of life, caused by the sudden loss of the stimulus of distention of the heart and bloodvessels, which do not readily contract down upon their contents.

2. The qualities of the blood are changed, from the previous discharge of some of its elements, while the others are retained.

*Fourth Stage.*—This stage consists essentially in fever of a low type, to which is often added local disease.

